# Neural response to trauma‐related and trauma‐unrelated negative stimuli in remitted and persistent pediatric post‐traumatic stress disorder

**DOI:** 10.1002/brb3.2173

**Published:** 2021-06-02

**Authors:** Peng Wang, Zu‐Lai Peng, Lu Liu, Li An, Yu‐Xin Liu, Qing‐Jiu Cao, Li Sun, Ning Ji, Yun Chen, Bin‐Rang Yang, Yu‐Feng Wang

**Affiliations:** ^1^ Sixth Hospital/Institute of Mental Health Peking University Beijing China; ^2^ National Clinical Research Center for Mental Disorders & The Key Laboratory of Mental Health Ministry of Health (Peking University) Beijing China; ^3^ Shenzhen Children's Hospital Shenzhen China; ^4^ Cardiac Rehabilitation Center, Fuwai Hospital CAMS&PUMC Beijing China

**Keywords:** child, earthquakes, hippocampus, post‐traumatic, remittance

## Abstract

**Introduction:**

Most youths who suffer from post‐traumatic stress disorder (PTSD) lose their diagnosis in the first 1–2 years. However, there are few studies on this brain mechanism, and the heterogeneity of the findings is partially due to the different stimuli applied and the mixed trauma history. Therefore, the use of trauma‐related/unrelated stimuli to study the remittance mechanism of earthquake‐induced PTSD could advance our knowledge of PTSD and inspire future treatment.

**Methods:**

Thirteen youths with PTSD, 18 remitted participants, and 18 control participants underwent functional magnetic resonance imaging (fMRI), while viewing trauma‐related pictures, trauma‐unrelated negative pictures, and scrambled pictures.

**Results:**

Under trauma‐unrelated condition, the neural activity of the left hippocampus in the remitted group was between the two other groups. Under trauma‐related condition, the PTSD and the remitted group exhibited higher neural activity in the right middle occipital gyrus than controls. The remitted group showed higher neural activity in the right parahippocampal gyrus and right lingual gyrus under trauma‐related condition than trauma‐unrelated condition, while no significant difference was found in PTSD group.

**Conclusion:**

PTSD status‐related group differences are mainly reflected in the left hippocampus under the trauma‐unrelated condition, while the hyperactivity in the right middle occipital gyrus under trauma‐related condition could be an endophenotype for PTSD.

## Graphical abstract

(a) Even after PTSD patients remitted, there are still PTSD‐like brain activities under trauma‐related condition; (b) In PTSD patients, specific brain activity under trauma‐related condition is generalized to trauma‐unrelated condition.

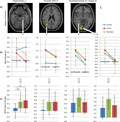

## 1 INTRODUCTION

Natural disasters always leave large numbers of young survivors suffering from various mental disorders, with post‐traumatic stress disorder (PTSD) being a most common one (Zhang et al., 2015). The prevalence of PTSD in youths after disasters ranges from 2.5% to 60.0% (Tang et al., 2017). Re‐experiencing/intrusion, avoidance/emotional numbing, cognition/mood negative alterations, and hypervigilance are the defining symptoms of PTSD (APA, 2013; Bovin et al., 2015). Most adolescents with PTSD recover spontaneously after an average of 14.8 months, but approximately one‐third show sustained psychopathology (McLaughlin et al., 2013). In a study of a Chinese population, the prevalence of PTSD in youths was 43.9% twelve months after an earthquake, and it dropped to 15.7% at the 30th month without treatment (Tang, Zhao, et al., 2017). The data from an adult study were similar (Rosellini et al., 2018). The brain mechanism of PTSD remission is important as to reveal the pathophysiology of PTSD and the development/improvement of interventions.

Research on the PTSD‐remittance mechanism has primarily focused on adults, while studies on youths are rare. Malejko et al. (2017) reviewed 19 longitudinal studies of PTSD (17 on adults and 2 on youths), and found that the remittance of PTSD was related to decreased activities of the insula and amygdala and increased activities of the prefrontal/anterior cingulate cortex (PFC/ACC) and hippocampus. In a serial of studies, Cisler et al. applied trauma‐unrelated emotional stimuli to female adolescents with PTSD and had several findings: (a) Adolescents with less symptom reduction were characterized by less threat‐safety discrimination before treatment (i.e., greater amygdala activation to both threat and neutral images), whereas adolescents with greater symptom reduction were characterized by amygdala activation only to threat images (Cisler et al., 2015); (b) The recovery of adolescent PTSD was positively related to the functional connectivity between the right amygdala and insula, and between the left amygdala and posterior cingulate gyrus (Cisler, Sigel, Steele, et al., 2016); and (c) The remittance of PTSD was related to the high modularity and assortativity of the whole‐brain network (Cisler, Sigel, Kramer, et al., 2016). Another study, which was performed on youths with PTSD due to interpersonal traumas and a control group without trauma experience, used a facial expression task (trauma‐unrelated) and found that the remittance was related to reduced activation of the posterior cingulate, mid‐cingulate and hippocampus (Garrett et al., 2019).

However, the heterogeneity existed in previous studies is one of the important concerns in this field (e.g., in the Malejko et al. (2017)'s review, differences in the hippocampus appeared in only three of the 19 reviewed studies, two of these three studies showed increased activation, and the other showed decreased activation). The frequently discussed possible factors that could contribute to the heterogeneity of findings include trauma history, stimulation paradigm (trauma‐related/unrelated), the control group (whether trauma exposed) (Malejko et al., 2017; Negreira & Abdallah, 2019), as well as scan effects, multiple comparisons (Chen et al., 2018), and complex analysis workflows (Botvinik‐Nezer et al., 2020), etc. The abnormal brain activity caused by various traumatic events was different (Boccia et al., 2016). The use of participants with the same trauma history may make the research more focused. Moreover, it also makes it possible to apply a unified trauma‐related stimulus across all participants, including trauma‐exposed controls. Most of the adult studies and all of the youth studies on PTSD‐remittance mechanisms used trauma‐unrelated stimuli (Garrett et al., 2019; Malejko et al., 2017). However, trauma‐related/unrelated stimuli should have different meanings. For example, van Rooij et al. (2016) used trauma‐unrelated emotional stimuli and found no difference in the hippocampus and ventromedial prefrontal cortex (vmPFC), which are often implicated in PTSD, between remitted and persistent PTSD veterans. This discrepancy may due to that these two regions were more involved in fear extinction recall (Rougemont‐Bucking et al., 2011). Trauma‐unrelated conditions cannot measure traumatic fear‐related processes, while trauma‐related stimuli would induce negative emotion and trauma‐specific fear in patients with PTSD (van Rooij et al., 2015). Presumably, the trauma‐specific component should be particularly meaningful in the PTSD‐remittance mechanism, because most effective therapies are trauma‐focused, such as cognitive‐behavioral therapy (CBT; Hinton et al., 2009), eye movement desensitization and reprocessing (EMDR; Mavranezouli, Megnin‐Viggars, et al., 2020; Mavranezouli, Megnin‐Viggars, et al., 2020) and prolonged exposure therapy (PE; Helpman et al., 2016; Maguen et al., 2019). To study this critical component for remittance, trauma‐related versus unrelated stimuli must be applied. However, so far no research used both conditions. A meta‐analysis found that trauma‐exposed controls and patients with PTSD exhibited hyperactivation of the amygdala, but no significant difference was found between these two groups, which indicates that this pattern of activation may not be pathological (Patel et al., 2012). Therefore, in studies using a trauma‐related stimulus, the use of traumatized controls may help to focus on the unique characteristics of PTSD rather than neural markers of trauma exposure.

The current functional magnetic resonance imaging (fMRI) experiment studied the unique remittance mechanism of pediatric PTSD via measurement of the corresponding neural activities of youths who remitted from PTSD, youths presenting persistent PTSD and traumatized controls 19 months after an earthquake, while their viewing earthquake pictures (trauma‐related) and trauma‐unrelated negative emotional pictures. We used whole‐brain analysis instead of predefined region of interests (ROI), because there are only a few related studies, with high heterogeneity. In addition, we also take into consideration that predefined ROIs may lead to overrepresentation in some brain regions [e.g., as suggested in both a recent review (Negreira & Abdallah, 2019) and a meta‐analysis (Sprooten et al., 2017) that amygdala activation is common only in studies using ROIs, but rarely in whole‐brain analysis]. Therefore, we hypothesized that, using whole‐brain analysis, under the trauma‐unrelated and trauma‐related conditions, the remitted group would show different brain activities from that of the PTSD group in the brain regions that related to remittance in previous studies: hippocampus/PCC/mPFC/amygdala; compared with the control group, patients with PTSD may have abnormal brain activities in the brain regions related to the classic pathological mechanism of PTSD: hippocampus/mPFC/amygdala; there would be no significant difference between the remitted group and control group. In trauma‐related versus. trauma‐unrelated contrast, the PTSD group may exhibit different brain activities, while there would be no significant differences in the remitted group.

## 2 METHODS

### 2.1 Participants

All participants were 8‐ to 18‐year‐old youths who survived the 2008 Wenchuan earthquake in China. Some of the behavioral data of these participants were included in our previous study (Yang et al., 2014). In the current fMRI experiment, psychiatric clinicians interviewed all participants according to the Present and Lifetime version of the Schedule for Affective Disorders and Schizophrenia for School‐Age Youth (K‐SADS‐PL; Kaufman et al., 2000) 17 months after the earthquake, but the fMRI scanning was applied at the 19th month because of the complicated preparation jobs before it. The following inclusion criteria were used for the PTSD group: (a) earthquake‐exposed youths; (b) diagnosed as current PTSD; and (c) right‐handed. The exclusion criteria were (a) other Axis‐I psychiatric diagnosis, except comorbid mood/anxiety disorders for the PTSD group; (b) IQ < 80 using the Chinese Wechsler Intelligence Scale for Children (C‐WISC; Gong & Cai, 1993); (c) use of psychotropic medications in the past 4 weeks; and (d) any significant medical or neurological conditions or a history of head injury. The inclusion and exclusion criteria of the remitted and control groups were similar to those of the PTSD group, except that the remitted participants met the diagnosis of lifetime PTSD, but not current PTSD in the 17th month interview, and that the controls never met the diagnosis criteria of any psychiatric diagnosis. Twenty‐five healthy controls, 15 subjects with PTSD and 23 remitted participants were recruited in our fMRI study. Data sets were also excluded for excessive head motion (translations and rotations were larger than 2.5 mm and 2.5 degrees) and poor accuracy on the task (over 10% missing). Participants included in the final analysis consisted of 18 healthy, 13 PTSD and 18 remitted youths. Among the 13 participants with PTSD, 2 of them received EMDR, 1 received EMDR and CBT, 1 received sertraline for 1 month, and 1 received clonidine controlled‐release patches 4 times. In the remitted group, 9 received EMDR, 2 received sertraline, 1 received sertraline and CBT, 1 received CBT, and 5 had spontaneous recovery. All EMDRs only lasted for 3 sessions, and CBTs for 8 sessions. According to the K‐SADS interview, none of the subjects had any other experience of traumatic stress events except this earthquake.

The Institutional Review Board at the Health Center of Peking University approved this study. Written informed consents were obtained from each participant and their guardians.

### 2.2 Affective processing task

During fMRI scan, participants performed a block‐designed affective processing task with earthquake‐related/unrelated emotional pictures and scrambled pictures as baseline. One trial consisted of a 4.5‐s picture and a 0.5‐s black screen. There were 6 trials in each block. The blocks were arranged in a fixed order: +S+N+E+S+N+E+S+N+E+ (+, rest; S, scrambled picture; N, trauma‐unrelated negative picture; E, trauma‐related earthquake picture). A relatively long rest time between blocks (20 s) was adopted to avoid anxiety elicited by the trauma pictures from persisting into the trauma‐unrelated pictures. All participants were asked to press the thumb button when they saw a picture appears to ensure that they were actually watching. Participants who did not respond to more than 10% of pictures were excluded.

**FIGURE 1:**
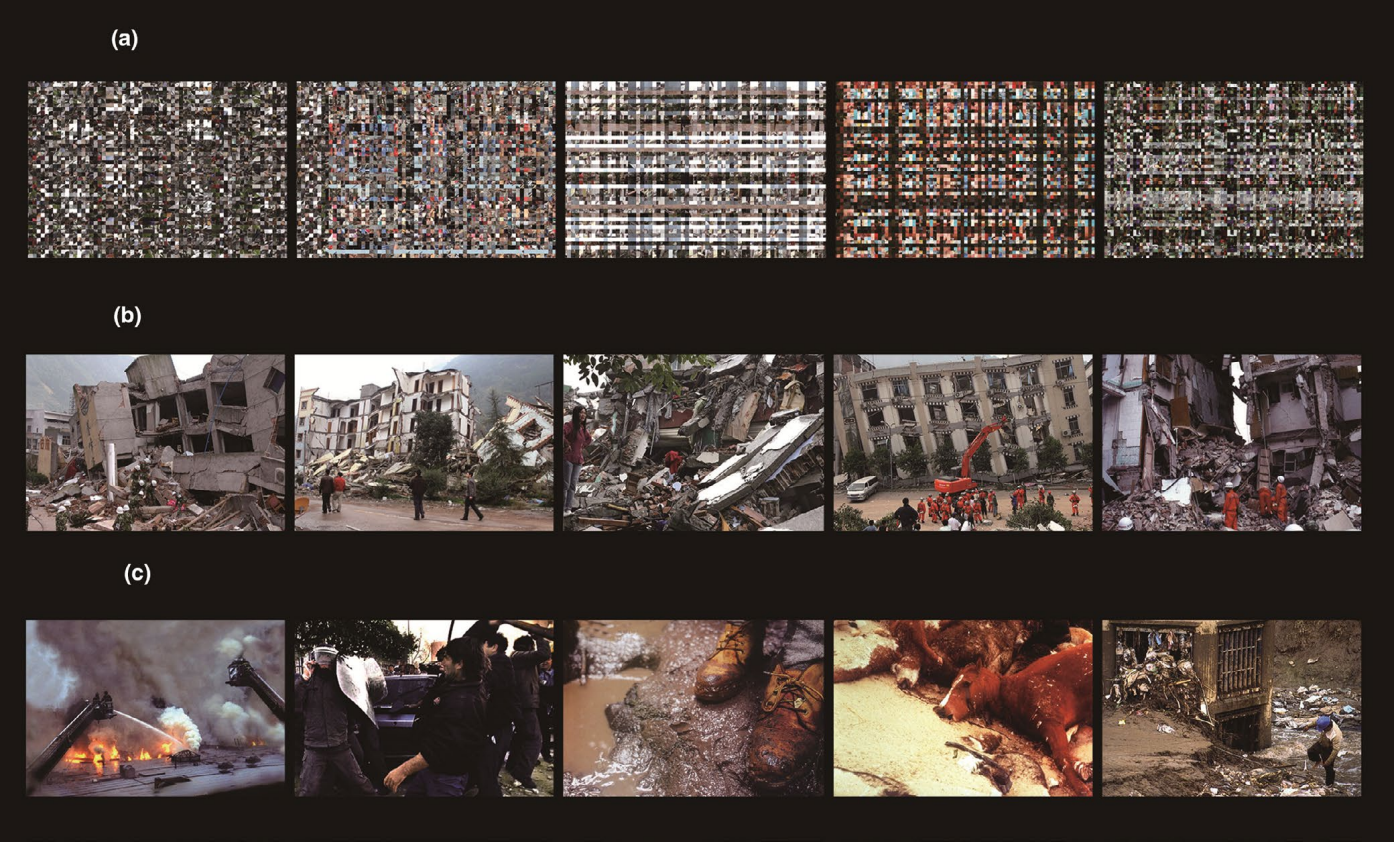
Example of stimulus pictures. (a) Scrambled pictures. (b) Trauma‐related pictures. (c) Trauma‐unrelated pictures

Eighteen pictures depicting the Wenchuan earthquake were collected from the Internet, and primarily portrayed collapsed buildings in Wenchuan with dead or wounded civilians. Eighteen negative but unrelated‐to‐earthquake‐scene pictures were selected from the International Affective Picture System [(IAPS; Jayaro et al., 2008), which has been widely used in PTSD research (Negreira & Abdallah, 2019)], and included depictions of diseases, poverty, filth, fire, violent assaults and horrible faces without any collapsed buildings. The two groups of pictures were balanced in emotional valence and arousal, as measured with a Self‐Assessment Manikin (SAM; Bynion & Feldner, 2017). Examples of the pictures are shown in Figure 1. Supplementary Material, which is available online, provides the details of the evaluation and screening of pictures, the making of scrambled pictures and the properties of images. Each group of pictures was randomly divided into three blocks.

### 2.3 fMRI data acquisition

Details were provided in the Supplementary Material.

### 2.4 fMRI data analysis

Preprocessing was performed using Statistical Parametric Mapping (SPM12, http://www.fil.ion.ucl.ac.uk/spm) and DPABI (a toolbox for Data Processing & Analysis for Brain Imaging; Yan et al., 2016; http://rfmri.org/DPABI). Additional details were provided in the Supplementary Material.

The experimental sequences (trauma pictures vs. scrambled pictures, trauma‐unrelated pictures vs. scrambled pictures, trauma pictures vs. trauma‐unrelated pictures) were modeled using a hemodynamic response function (HRF)‐convolved boxcar model with no derivatives, and global scaling was applied. We first produced between‐condition SPMs for each participant. Second, we undertook between‐condition contrasts at a within‐group level and a between‐group level. Chen et al. (2018) compared several multiple comparison correction strategies with respect to family‐wise error rate (FWER), and recommended the use of Gaussian random field (GRF) correction. In the within‐group contrasts, voxels of significant activity (trauma‐related vs. scrambled, trauma‐unrelated vs. scrambled, voxel thresholds *p* < .0001, cluster thresholds *p* < .005, one‐tailed) in all three groups, and voxels with significant different reaction to trauma‐related/unrelated stimuli (trauma‐related vs. trauma‐unrelated, voxel thresholds *p* < .0001, cluster thresholds *p* < .005, two‐tailed) in the PTSD and remitted groups were determined after thresholding using GRF correction in the DPABI toolbox. To compare differences in brain activity between the three groups under trauma‐related/unrelated conditions, the between‐group contrasts were applied in two conditions separately. We generated a union mask of activated brain regions (*p* < .05, no correction) in any group and performed ANOVA (analysis of variance) within the mask with GRF correction (voxel thresholds *p* < .005, cluster thresholds *p* < .05). Given that age may significantly moderate the effect of stressful events on brain function (Pechtel & Pizzagalli, 2011), we used age as a covariate in the comparison between groups. Considering that gender/comorbidity may have impact too, we also tried to use age + gender + comorbidity as covariates. In post hoc analysis, we extracted values from resulting clusters that showed a main effect of group in the ANOVA for each participant and analyzed between‐group differences with independent samples *t* tests.

We also explored the group analysis and within‐group analysis (in the control group) on the trauma‐related versus trauma‐unrelated contrast, as well as correlation analysis in brain regions to study the relationship between brain activities.

## 3 RESULTS

### 3.1 Demographic data

The information for the demographics/clinical data of the participants is summarized in Table 1. The differences in gender, age, and IQ were not significant.

**TABLE 1:** Demographics/clinical information of participants

Group	Healthy	PTSD	Remitted	Statistics	
	(*n* = 18)	(*n* = 13)	(*n* = 18)	*F/χ* ^2^	*p*
Age (years)	15.21 (1.63)	15.21 (2.50)	15.23 (1.93)	*F* (2,46) = 0.001	0.999
	Range: 12.1–18.1	Range: 8.9–18.1	Range: 10.4–18.3		
Gender (M:F)	10:8	4:9	6:12	*χ^2^* = 2.579	0.275
IQ	106.67 (12.00)	108.85 (14.46)	102.39 (10.46)	*F* (2,46) = 1.156	0.324
	Range: 80–125	Range: 83–129	Range: 84–127		
Comorbidities	None	3 MDD	1 MDD		<0.001

Abbreviations: major depressive disorder; MDD; PTSD, post‐traumatic stress disorder.

### 3.2 Trauma‐unrelated brain responses in the three groups

**FIGURE 2:**
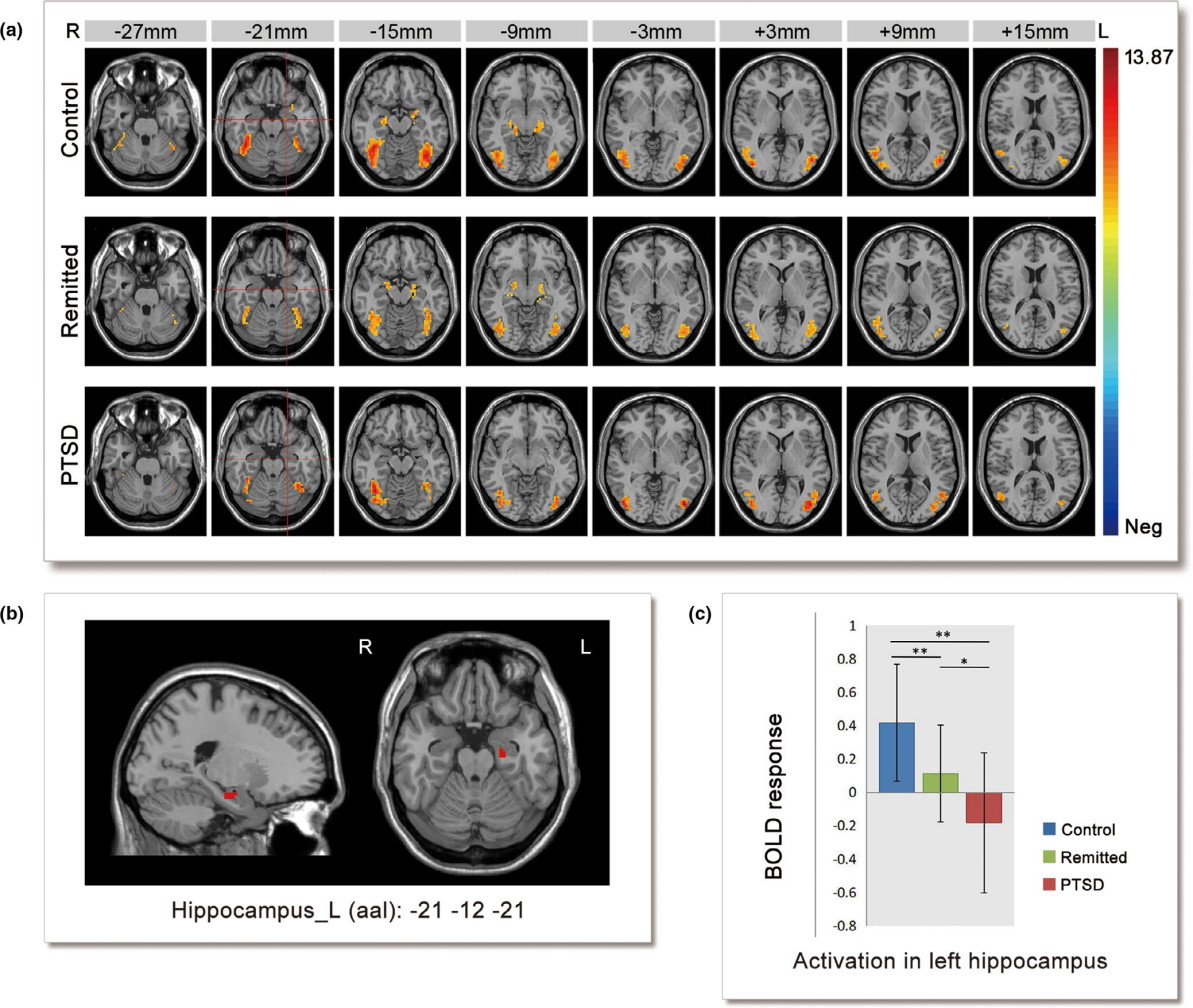
Neural activity differences between groups under the trauma‐unrelated condition. (a) Trauma‐unrelated > scrambled activation in the three groups, threshold set at whole‐brain GRF correction voxel thresholds *p* < .0001, cluster thresholds *p* <. 005, one‐tailed. (b) Left hippocampus cluster, ANOVA results for the (trauma‐unrelated pictures vs. scrambled pictures) contrast within a binary mask by union the three within‐group SPMs, with threshold using GRF correction voxel thresholds *p* <. 005, cluster thresholds *p* <. 05. *k* = 19, Peak *xyz* = −21 –12 –21 (shown in (a) with red cross). (c) The activities of the three groups in the left hippocampus gyrus cluster. PTSD: post‐traumatic stress disorder, BOLD = blood oxygenation level‐dependent, *p < .05, **p <. 01

Activation patterns for each group under the trauma‐unrelated condition are shown in Figure 2a. The significantly activated cluster was primarily located in the bilateral middle occipital gyrus. The control and remitted groups showed activated bilateral hippocampus, and the PTSD group showed no significant activation in that area. ANOVA revealed that the brain region located in the left hippocampus/parahippocampus (peak *xyz* = −21 –12 –21; *k* = 19 voxels) (Figure 2b) exhibited a significant main effect of group. However, when we tried a relatively strict correction (GRF correction, voxel thresholds *p* < .005, cluster thresholds *p* < .005), this result was not significant. Post hoc analysis showed that the brain activity of this cluster in the remitted group was higher than in the PTSD group and lower than in the controls (PTSD < remitted < controls). (Figure 2c). When age/age + gender + comorbidity were used as covariates in ANOVA, the cluster in the left hippocampus was still significant, even after the stricter correction (Figure S1).

### 3.3 Trauma‐related brain responses in the three groups

**FIGURE 3:**
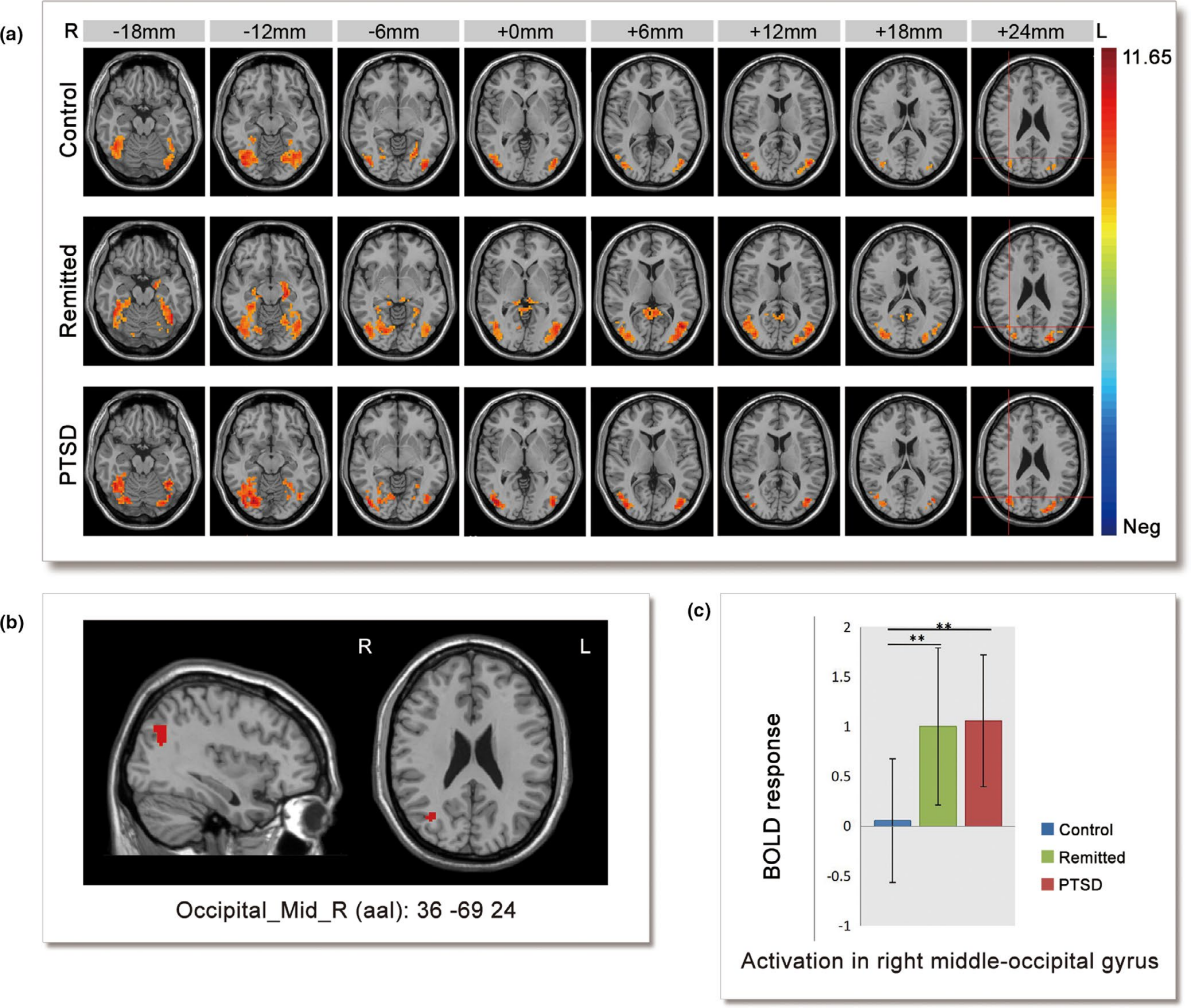
Neural activity differences between groups under trauma‐related conditions. (a) Trauma‐related > scrambled activation in the three groups, threshold set at whole‐brain GRF correction voxel thresholds *p* < .0001, cluster thresholds *p* < .005, one‐tailed. (b) Right middle occipital gyrus cluster, ANOVA results for the (trauma‐related pictures vs. scrambled pictures) contrast within a binary mask by union of the three within‐group SPMs, with a threshold using GRF correction voxel thresholds *p* < .005, cluster thresholds *p* < .05. *k* = 40, Peak *xyz* = 36 –69 24 (shown in (a) with red cross). (c) The activities of the three groups in the right middle occipital gyrus cluster. PTSD: post‐traumatic stress disorder, BOLD = blood oxygenation level‐dependent, **p < .01

Activation patterns for each group under trauma‐related conditions are shown in Figure 3a. The patterns of the three groups were similar and included the bilateral occipital cortex, primarily the middle occipital gyrus and lingual gyrus, extending to the bilateral temporal lobe and bilateral parahippocampal gyrus. ANOVA revealed that the brain area that exhibited a significant main effect of group was located in the right middle occipital gyrus/precuneus (peak *xyz* = 36 –69 24, *k* = 40 voxels) (Figure 3b). This result was still significant when a relatively strict threshold was applied (GRF correction, voxel thresholds *p* < .005, cluster thresholds *p* < .005). Post hoc analysis showed that brain activity in the PTSD group and remitted group was significantly higher than in the control group, but there was no difference between the PTSD and remitted groups (controls < PTSD = remitted) (Figure 3c). When age/age + gender + comorbidity were used as covariates, this cluster was also significant. (Figure S2). When we tried to use the cluster in the left hippocampus from the previous analysis (Figure 2b) as a mask for ANOVA, there was no significant difference among the three groups (GRF correction, voxel thresholds *p* < .05, cluster thresholds *p* < .05).

### 3.4 Neural activity difference between trauma‐related/unrelated conditions

#### 3.4.1 PTSD group: trauma‐related versus. trauma‐unrelated

Surprisingly, we found no significant neural activity difference between the two conditions in the PTSD group after GRF correction (voxel thresholds *p* < .0001, cluster thresholds *p* < .005, two‐tailed). There was still no significant result when we tried a relatively loose threshold (*p* < .001, cluster thresholds *p* < .05, two‐tailed).

#### 3.4.2 Remitted group: trauma‐related versus trauma‐unrelated

**FIGURE 4:**
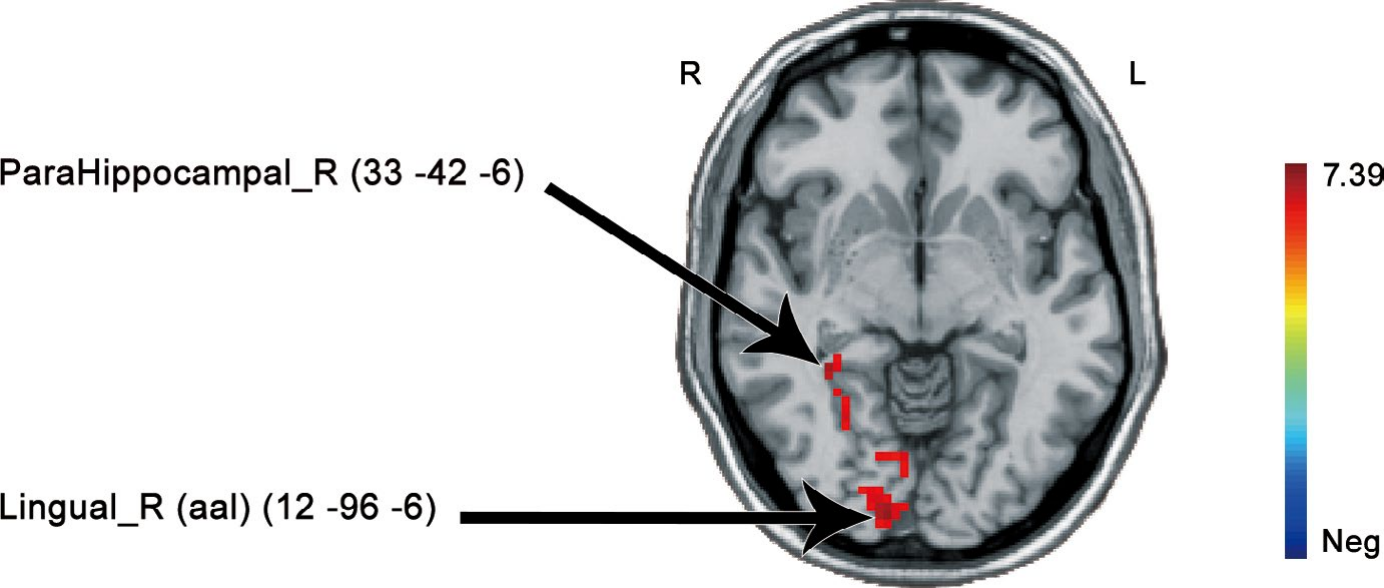
Neural activity differences in trauma‐related > trauma‐unrelated contrast in the remitted group. Upper cluster *k* = 43, Peak *xyz* = 33 –42 –6; Lower cluster *k* = 97, Peak *xyz* = 12 –96 –6; threshold set at whole‐brain GRF correction voxel thresholds *p* < .0001, cluster thresholds *p* < .005, two‐tailed

In the remitted group, trauma‐specific pictures elicited a significant increase in neural activity in the right parahippocampal gyrus (peak *xyz* = 33 –42 –6; *t* = 7.01; *k* = 43 voxels) and right lingual gyrus (peak *xyz* = 12 –96 –6; *t* = 7.39; *k* = 97 voxels) compared with the trauma‐unrelated condition (Figure 4).

### 3.5 Exploratory analysis

#### 3.5.1 Between‐group comparison in trauma‐related/unrelated contrast

**FIGURE 5:**
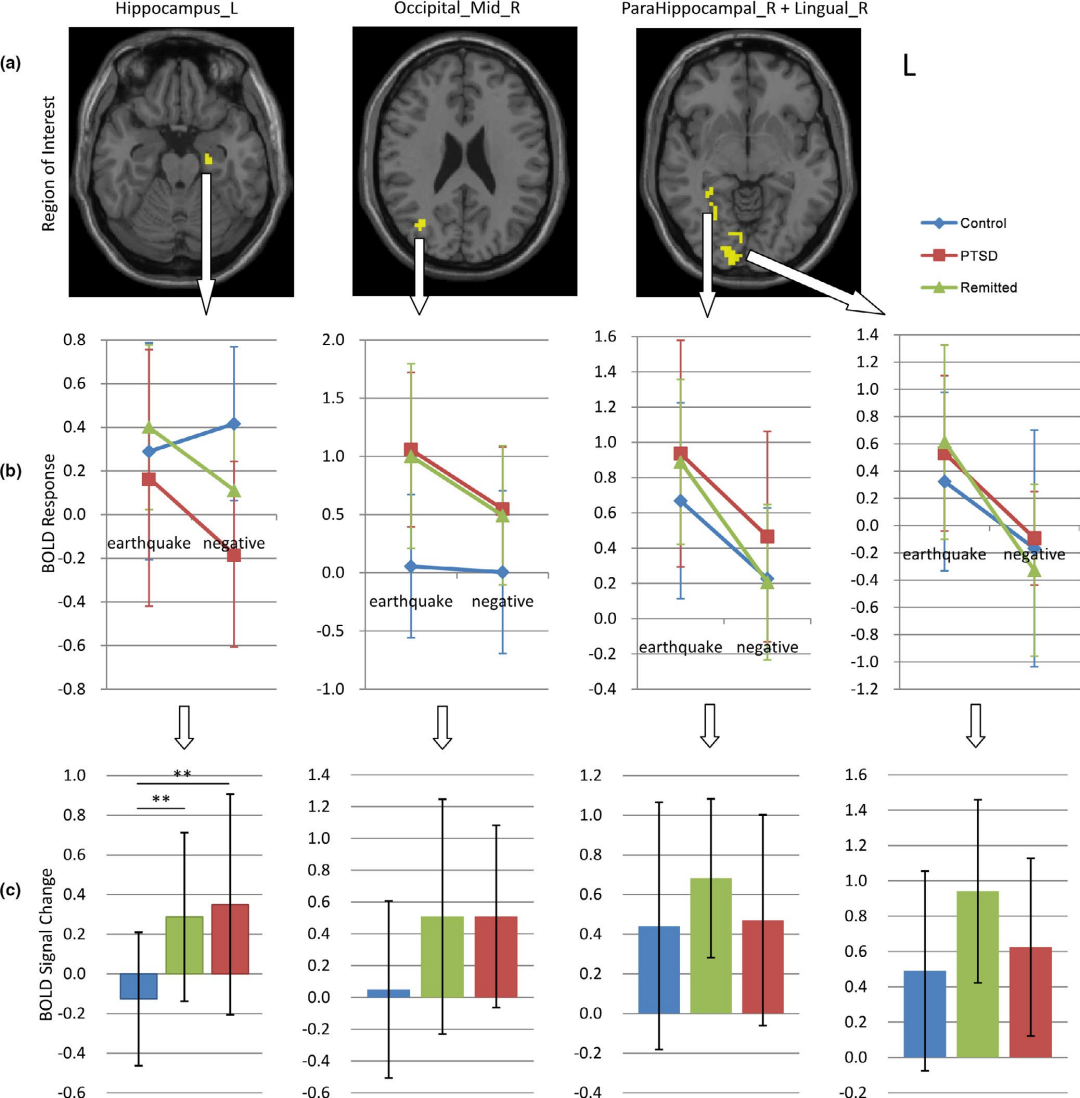
Neural activity in four clusters under trauma‐related and trauma‐unrelated conditions. (a) The location of four clusters. (b) The neural activity in three groups under trauma‐related (earthquake) and trauma‐unrelated (negative) conditions. (c) Between‐group comparisons of signal change between conditions. PTSD: post‐traumatic stress disorder, BOLD = blood oxygenation level dependent, **p < .01

No significant group effect was found in the whole‐brain analysis (voxel thresholds *p* < .005, cluster thresholds *p* < .05). When we conducted analysis in the mask of four brain regions with positive results in the above analysis separately (left hippocampus, right middle occipital gyrus, right parahippocampal gyrus and right lingual gyrus), we found that only the left hippocampus survived after correction (GRF correction voxel *p* < .0005, cluster *p* < .0005, post hoc: controls < remitted = PTSD). To visually show the relative and absolute differences of brain activity in three groups under different conditions, we show the signals extracted from these clusters, as well as the results of the between‐group comparison (Figure 5). When age was applied as a covariate, the left hippocampus survived after GRF correction (voxel *p* < .001, cluster *p* < .0005) but did not pass a stricter correction; the other results were the same as no covariate. When age + gender + comorbidity was used as covariates, the results were the same as no covariate (Figure S3).

#### 3.5.2 Control group: trauma‐related versus trauma‐unrelated

See Supplementary Material and Figure S4.

#### 3.5.3 Correlation between left hippocampus brain activities under the trauma‐unrelated condition and signal change between conditions

See Supplementary Material.

## 4 DISCUSSION

The current study focused on the brain mechanism of remitted PTSD children and adolescents after earthquake compared with current PTSD and trauma‐exposed controls using trauma‐unrelated and trauma‐related stimuli. As hypothesized, in the comparison between the PTSD group and the remitted group, under the trauma‐unrelated condition PTSD exhibited lower activity in the left hippocampus; however, no significant difference was found under the trauma‐related condition. In the comparison between the PTSD group and the controls, PTSD exhibited lower activity in the left hippocampus under the trauma‐unrelated condition and higher activity in the right middle occipital gyrus under the trauma‐related condition. There was a similar pattern in the comparison between the remitted group and the control group, but the activity of the left hippocampus of the remitted group was between the other groups under the trauma‐unrelated condition. In trauma‐related versus trauma‐unrelated contrast, contrary to our hypothesis, there was no significant neural activity difference in the PTSD group, while in the remitted group, different brain activities were found in the right parahippocampal gyrus and right lingual gyrus. We also explored a group analysis of trauma‐related versus trauma‐unrelated contrast and found that the activity difference in the left hippocampus was PTSD = remitted > controls. No difference in mPFC/ACC/amygdala was found in any comparison.

The pathological mechanism of PTSD is closely related to the hippocampus. The largest PTSD brain imaging study, which included 1,868 participants from 16 cohorts, confirmed the relationship between PTSD and a smaller hippocampus volume (Logue et al., 2018). Klaming et al. (2019) also found the correlation between right hippocampus morphology and symptom severity in 70 trauma‐exposed veterans. Furthermore, Malejko et al. (2017) reviewed the researches on PTSD remission and found that the remission of PTSD was often accompanied by a change in hippocampus activity. In the current study, using whole‐brain analysis, the only significant difference between the PTSD group and the remitted group was in the left hippocampus under the trauma‐unrelated condition. To be specific, the remitted group exhibited higher activity than the PTSD group, and lower than the control group. This finding supported the significance of the hippocampus, as well as trauma‐unrelated stimuli, in the remittance mechanism of PTSD. In the following exploratory analysis, we also found that this cluster was the only one with between‐group differences under trauma‐related versus trauma‐unrelated contrast.

A number of studies have found that the volume of the occipital lobe in the PTSD population is smaller than that of the control group (Cwik et al., 2019; Sussman et al., 2016). In adult (Gudrun et al., 2013; Ke et al., 2015) and children (Yang et al., 2004) PTSD studies, this region also showed abnormal high activity under trauma‐related conditions. This may be due to the involvement of the occipital lobe in the transformation of traumatic memory in visual form into narrative trauma‐related memories (Lanius et al., 2006). In the current study, the hyperactivity in right middle occipital gyrus in the PTSD and remitted group may reflect the re‐experiencing under trauma‐related stimuli condition.

Contrary to our intuition, the remitted individuals still had PTSD‐like brain responses to trauma stimuli. This state‐independent feature may be interpreted as the endophenotype of PTSD (McAuley et al., 2014). However, there is still a question from the other side of this phenomenon: how can we define an individual who still exhibits “abnormal” brain activity under trauma stimuli as remitted? This dichotomy may occur because our definition of the state of psychiatric disorder highly depends on the influence or potential influence of symptoms on an individual's social functioning, and the “abnormal” brain activity that existed only during trauma‐specific stimulation had relatively little influence on it. The PTSD diagnostic criterion G in DSM‐5 is “The disturbance causes clinically significant distress or impairment in social, occupational, or other important areas of functioning” (APA, 2013), which directly points to the evaluation of social functions (Bovin et al., 2015). If an individual only have “abnormal response” to stimuli that are highly specific to the original trauma (trauma‐related stimulus), the chance of exposure in daily life to cause functional damage will be relatively low, and the patient will consequently be defined as ‘remitted,” even if the possibility of an abnormal response under very special circumstances exists. Schnurr and Lunney (2016) studied the relationship between symptom improvement benchmarks and the quality of life of patients with PTSD and found that remission (defined as loss of diagnosis plus a severity score <20 in the Clinician‐Administered PTSD Scale) did not yield more benefit than the loss of diagnosis, although remission is considered the most desirable outcome for relieving PTSD symptom burden. The loss of diagnosis in patients with PTSD does not always mean complete “normalization” at the symptom level, but more meaningfully at the level of social functioning. Similarly, in the current study, participants who were defined as remitted did not achieve complete “normalization” in neural activities. There could be a potential variable related to social functioning of earthquake‐induced PTSD—the “Commonness” of the trauma, referring to the probability of exposure to related cues in daily life. In the current study, earthquake‐related cues should be relatively rare in daily life, and correspondingly, the chance to affect individuals was low. Frequent stimulation does cause pain to individuals, but also provides more opportunities for extinction training.

In the remitted group, the right parahippocampal gyrus and the right lingual gyrus exhibited higher brain activity under the trauma‐related condition than under the trauma‐unrelated condition. These clusters were highly consistent with the findings from another earthquake‐induced PTSD study on youths, in which PTSD youths exhibited higher activity than controls in the same brain regions (Yang et al., 2004). The parahippocampal gyrus is involved in the storage and retrieval of emotional memory (Yang et al., 2004), while the lingual gyrus is related to narrative memory (Lanius et al., 2006). In previous study, reduction in the lingual gyrus is related to the severity of PTSD symptoms (Wrocklage et al., 2017). The current findings in the remitted group may also reflect the re‐experiencing under trauma condition, and there is no such response under the trauma‐unrelated condition.

In contrast, there is no significant brain activity difference in the PTSD group between the two conditions. This result, however, needs to be discussed very carefully. We previously considered whether this statistically negative result could be a supportive evidence for the claim in previous studies that abnormalities of patients with PTSD are “generalized” to trauma‐unrelated condition (van Rooij et al., 2015; Zinchenko et al., 2017). However, inspection of Figure 5 shows that this trauma related versus trauma‐unrelated difference between the groups is rather small and was not statistically significant. Exploratory correlation analysis also failed to find a significant correlation between brain activity and generalization effect. In the future, bigger sample with better statistical power will be necessary to be applied to verify these findings. In addition, if researchers want to study the generalization effect, a more specific material should be considered, such as hieratically larger circles, to get a more convincing conclusion.

The current findings support that trauma‐unrelated negative stimuli, rather than trauma stimuli, play an important role in PTSD remission. Though most effective therapies for PTSD are trauma‐focused (Helpman et al., 2016; Hinton et al., 2009; Maguen et al., 2019; Mavranezouli, Megnin‐Viggars, et al., 2020; Mavranezouli, Megnin‐Viggars, et al., 2020), nontrauma ‐focused interventions (such as Dialectical Behavioural Therapy, Yoga, and art therapy) are emerging (Racco & Vis, 2015). Kaczkurkin et al. (2017) argued that because of the important role of maladaptive generalization in the pathological mechanism of PTSD, future psychotherapy may also involve trauma‐like stimulation (generalized stimulation). Therefore, negative emotional stimuli (with high commonness) must be taken into account.

We did not find differences between groups in PFC/ACC or amygdala, which were reported in previous PTSD‐remittance studies (Cisler et al., 2015; Negreira & Abdallah, 2019). This discrepancy may be caused by differences in age, gender, trauma experience, use of ROI, and the paradigms applied (Malejko et al., 2017; Negreira & Abdallah, 2019). The classic pathological model of PTSD is mostly based on adult studies. However, there should be differences between youths and adults (Herringa, 2017). Given the age span of the subjects in the present study was very large, from 8 to 18, it might have contributed to this “null” finding. However, the covariate analyses denied its contribution. A previous PTSD study using trauma‐related stimulus found different brain activities in the amygdala and ACC in different genders (Shin et al., 2004). The mixed genders in the current study may erase the effect of some brain regions, especially the amygdala and mPFC. In addition, considering the special original trauma (earthquake) in the current study, and thus differences in paradigm/stimulus, the results will also be affected. Interestingly, the brain regions revealed by the current study are similar to the only PTSD study on mixed gender youths after an earthquake, which included occipital lobe, hippocampus/parahippocampus, but no prefrontal lobe/ACC (Yang et al., 2004).

Our study had several limitations. First, a small sample size was used due to the common difficulties of task‐state fMRI studies on natural disaster‐induced PTSD. Among all 10 previous related studies, the sample sizes of the PTSD groups ranged from 5 to 16 subjects, with an average of 10.9 subjects (Du et al., 2014; Piccardi et al., 2016; Yang et al., 2004). Small groups provide relatively low statistical power and make it difficult to strictly control the comorbidity and treatment of subjects. Second, the statistical thresholding was liberal, which increased the risk for false‐positive results. However, it also makes our negative findings more robust, such as the similarity of brain activities between the remitted and PTSD groups under trauma‐related conditions. In addition, the *p* value of GRF correction in this study is less than 0.01, which is enough to make the false‐positive rate lower than 0.05, even if the spatial distribution does not satisfy the Gaussian distribution (Eklund et al., 2016). Third, the generalizability of the current findings may be limited because of the particularity of earthquake‐induced PTSD. Previous studies showed that the type of original trauma affected the heterogeneity of PTSD research (Boccia et al., 2016; Negreira & Abdallah, 2019). The low “Commonness” of earthquake‐specific stimuli may also have a potential impact on the remittance processing of patients with PTSD. Future PTSD experiments should measure the “Commonness” of stimuli and study their influence on PTSD. It was a pity we had neither asked the participants to evaluate the pictures after the scanning, nor required them to describe their feelings and thoughts during the process. Future researcher had better take these factors into consideration.

## 5 CONCLUSION

This study investigated the brain mechanisms of PTSD remittance using two kinds of stimuli and found that PTSD symptom‐related group difference is mainly reflected in the left hippocampus under the trauma‐unrelated condition, while the hyperactivity in the right middle occipital gyrus under trauma‐related condition could be an endophenotype for PTSD.

## CONFLICT OF INTEREST

The authors declare that they have no competing interests.

## Supporting information

Supplementary Material

Fig S1

Fig S2

Fig S3

Fig S4

## Data Availability

The datasets during and/or analyzed during the current study available from the corresponding author on reasonable request.
