# Supplementary material for: Neural response to trauma‐related and trauma‐unrelated negative stimuli in remitted and persistent pediatric post‐traumatic stress disorder
Source: Brain Behav. 2021 Jun 2;11(7):e02173. doi: 10.1002/brb3.2173 (PMC8323042; doi:10.1002/brb3.2173)
Supplement: Supplementary file 1 — Supplementary Material [file BRB3-11-e02173-s002.docx]

**Supplementary Material for Review**

**Picture stimulation preparation**

Seventy-two pictures depicting the Wenchuan earthquake were collected from the internet and primarily portrayed collapsed buildings in Wenchuan with dead or wounded civilians. Seventy-two negative but unrelated-to-earthquake-scene pictures were selected from the International Affective Picture System [(IAPS;Jayaro, de la Vega, Diaz-Marsa, Montes, & Carrasco, 2008), which is widely used in PTSD research (Negreira & Abdallah, 2019)] and included depictions of diseases, poverty, filth, fire, violent assaults and horrible faces without any collapsed buildings. All pictures were modified to 800*600 pixels with 72 dpi resolution. Forty-five healthy youths who also experienced this earthquake (but were not included in the later scanning) were evaluated for their affective response (emotional valence and arousal) to these pictures using the Self-Assessment Manikin (SAM) and a 7-point Likert Scale (0 = ‘not unpleasant’ to 6 = ‘very unpleasant’ and 0= ‘not arousing’ to 6 = ‘very arousing’). The Cronbach’s alpha values of the 45 judges’ ratings was 0.92 for valence and 0.90 for arousal. The estimated reliabilities of the average individual judges (mean Pearson r) were 0.67 for valence ratings and 0.61 for arousal ratings (*p* < 0.01).

Eighteen trauma-related earthquake pictures and 18 trauma-unrelated negative pictures with valence and arousal in the same range (valance 3.88~2.87, arousal 3.88~2.59) were selected for the fMRI task. Eighteen digital scrambled pictures were created out of the 18 earthquake pictures.

**fMRI Data acquisition**

Neuroimage data were acquired at a SIEMENS TRIO 3-Tesla scanner (Siemens, Erlangen, Germany) at Huaxi MR Research Center, West China Hospital of Sichuan University. Echo-planar images depicting blood-oxygen-level-dependent (BOLD) contrast were collected under the following conditions: 30 axial slices of 5 mm thick with no gap; repetition time (TR): 2000 ms; echo time (TE): 30 ms; flip angle: 90°; field of view (FOV): 240×240 mm; matrix = 64 ×64. High-resolution T1-weighted images were simultaneously obtained from each participant for anatomical reference under the following conditions: 176 sagittal slices of 1 mm thick; TR: 1.9s, TE: 2.26 ms; inversion time (TI): 900 ms; flip angle: 9º; FOV: 256×256 mm.

**Preprocessing of fMRI data**

Eight time points were discarded (because of 2 dummy TRs before the scanning, the picture block began at the 9th time point). Corrections were made for motion artifacts of the participants, and the images were spatially normalized into standardized Montreal Neurological Institute (MNI) anatomical space by using T1 unified segmentation. Registrations were visually inspected for accuracy. After smoothing with a Gaussian kernel (full-width half-maximum [FWHM]: 4 mm), statistical parametric mapping analysis was performed using SPM12.

**Between-Group Comparison Under Trauma-Unrelated vs. Scramble Contrast with Covariates, Supplement Figure 1.**


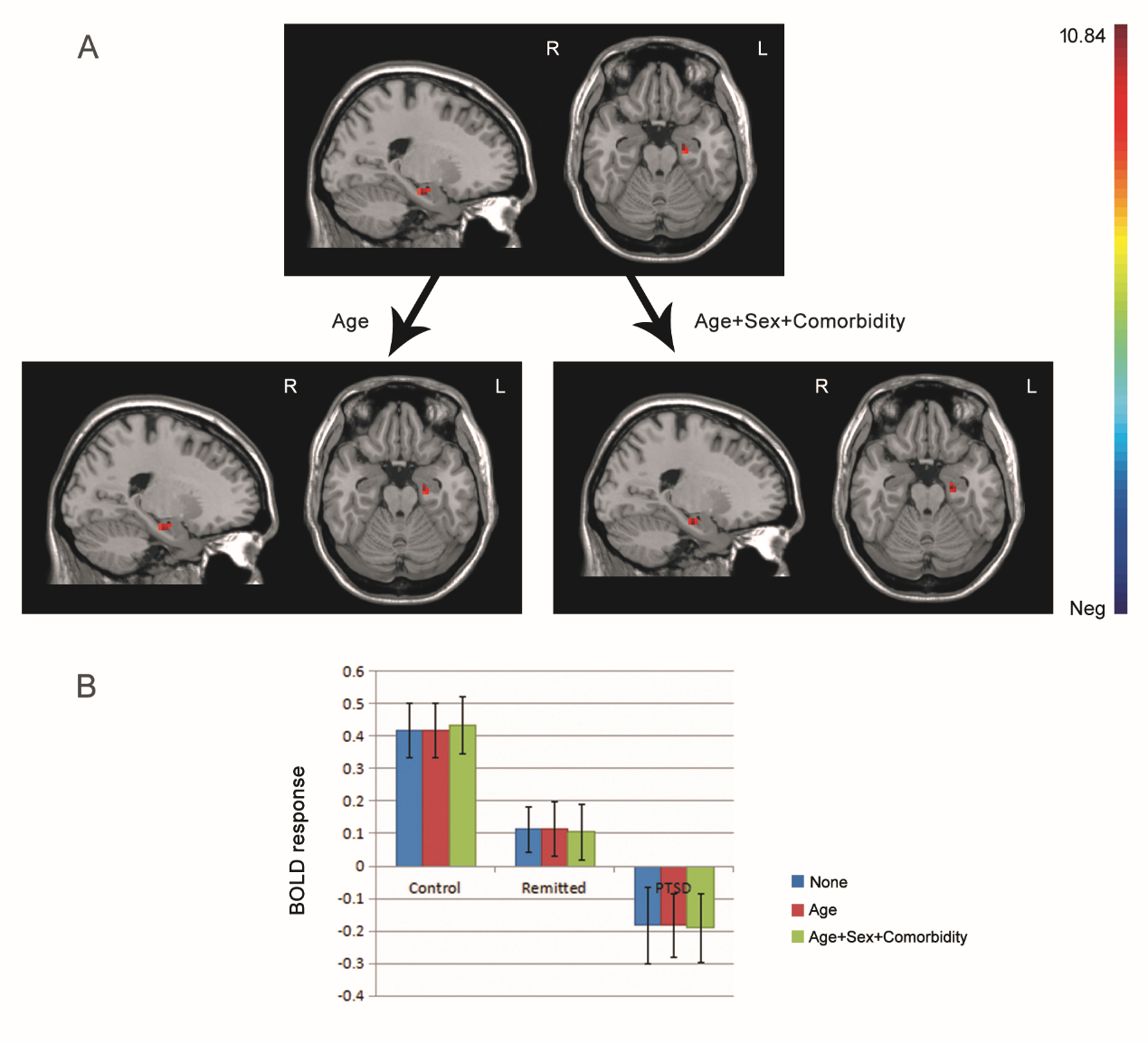


Supplement Figure 1. The results of ANOVA with age/age+gender+comorbidity as covariates among the three groups under the trauma-unrelated condition. (A) Shows the cluster in the left hippocampus, which is still significant after covariate analysis (GRF-corrected voxel *p* < 0.005, cluster *p* < 0.005). (B) Visual representation of the extracted signal.

**Between-Group Comparison Under Trauma-Related vs. Scramble Contrast with Covariates, Supplement Figure 2.**


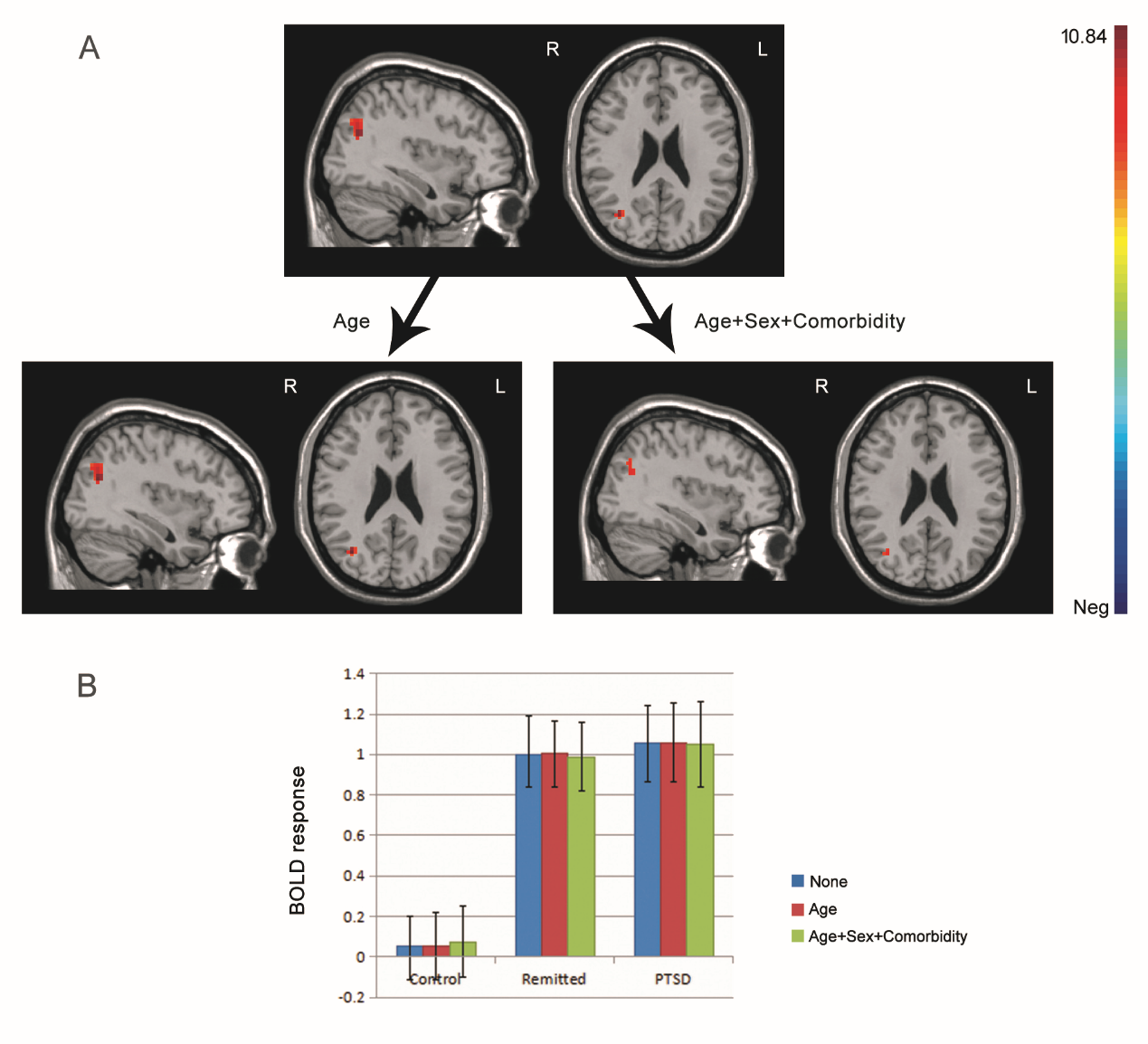


Supplement Figure 2. The results of ANOVA with age/age+gender+comorbidity as covariates among the three groups under the trauma-related condition. (A) Shows the cluster in the right occipital lobe, which is still significant after covariate analysis (GRF-corrected voxel *p* < 0.005, cluster *p* < 0.005). (B) Visual representation of the extracted signal.

**Between-Group Comparison Under Earthquake vs. Negative Contrast with Covariates, Supplement Figure 3.**


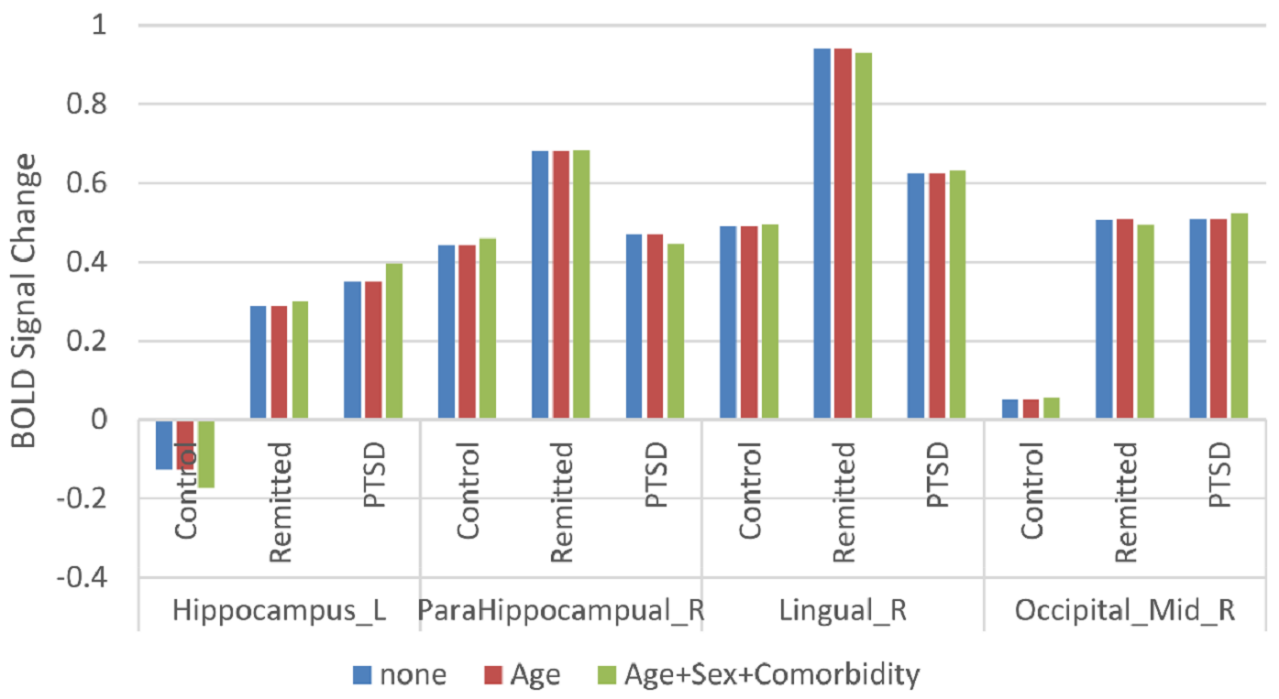


Supplement Figure 3. Group analysis of the trauma-related vs trauma-unrelated contrast with covariate. The four parts from left to right are the brain activity in the mask of left hippocampus, right parahippocampal gyrus, right lingual gyrus and right middle occipital gyrus. When age was applied as a covariate, the left hippocampus is still significant after GRF correction (voxel *p* < 0.001, cluster *p* < 0.0005) .

**Control Group: Trauma-Related vs. Trauma-Unrelated**

To explore the possible non-pathological differences in brain activity in the control group, we compared the trauma-related vs. trauma-unrelated conditions in the whole brain, and trauma < trauma-unrelated brain activity was shown in the bilateral cerebellum (GRF-corrected voxel *p* < 0.0001, cluster *p* < 0.005, two-tailed) (Supplement figure 4).


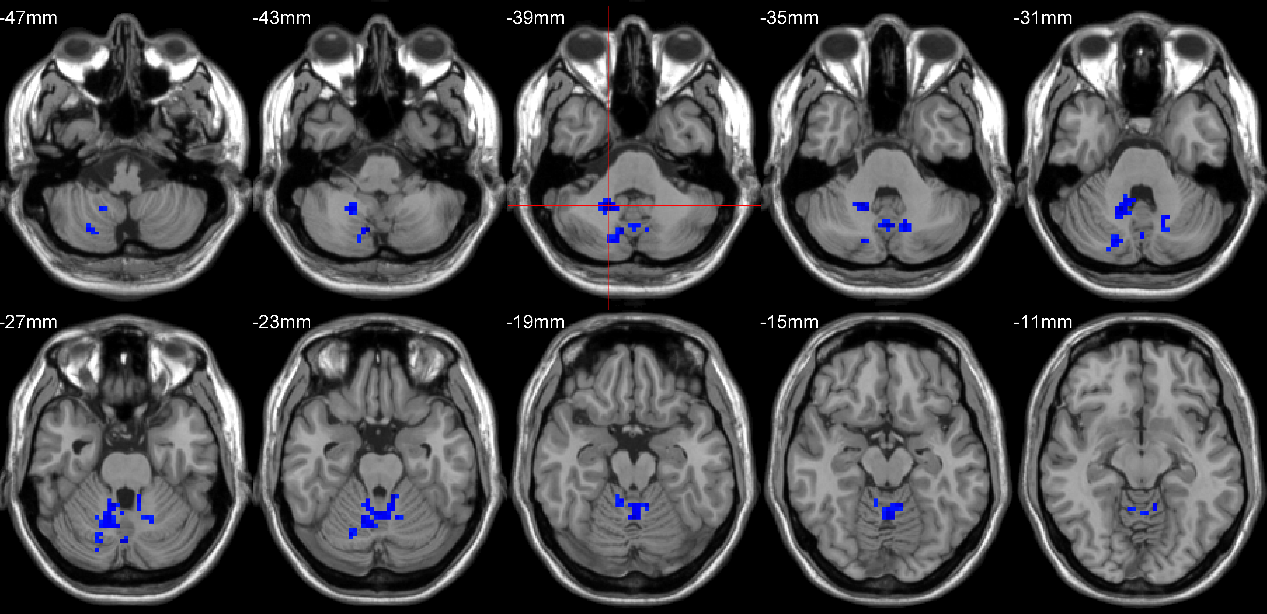


Supplement figure 4. Trauma < trauma-unrelated brain activity in the trauma-related vs trauma-unrelated contrast in the control group.

**Correlation Between Left Hippocampus Brain Activities under the Trauma-Unrelated Condition and Signal Change Between Conditions**

We explored the relationship between brain activity in the left hippocampus (which plays an important role in discrimination function and showed a significant difference between groups under the trauma-unrelated condition) under the trauma-unrelated condition, and the brain activity difference under trauma-related vs. trauma-unrelated contrast (which could stand for "successful discrimination"). By correlation analysis, we did not find any significant correlation between them in the right parahippocampal gyrus or right lingual gyrus (*p* > 0.05) in the three groups.

References

Jayaro, C., de la Vega, I., Diaz-Marsa, M., Montes, A., & Carrasco, J. L. (2008). [The use of the International Affective Picture System for the study of affective dysregulation in mental disorders]. *Actas Esp Psiquiatr, 36*(3), 177-182.

Negreira, A. M., & Abdallah, C. G. (2019). A Review of fMRI Affective Processing Paradigms Used in the Neurobiological Study of Posttraumatic Stress Disorder. *Chronic Stress (Thousand Oaks), 3*. doi:10.1177/2470547019829035
