# Supplementary figures and images for: Neural response to trauma‐related and trauma‐unrelated negative stimuli in remitted and persistent pediatric post‐traumatic stress disorder
Source: Brain Behav. 2021 Jun 2;11(7):e02173. doi: 10.1002/brb3.2173 (PMC8323042; doi:10.1002/brb3.2173)

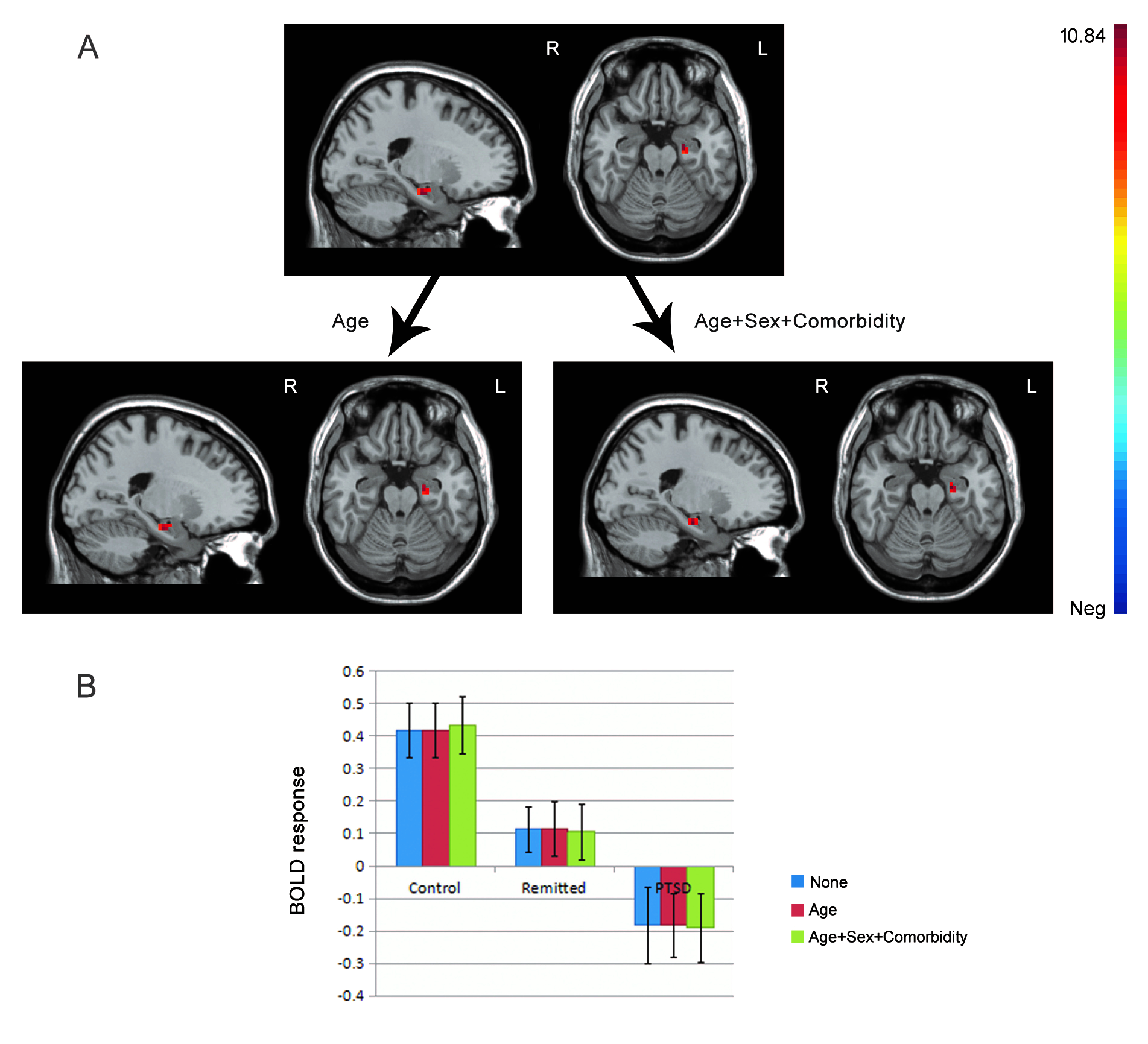

Supplement: Supplementary file 2 — Fig S1 [file BRB3-11-e02173-s003.tif]

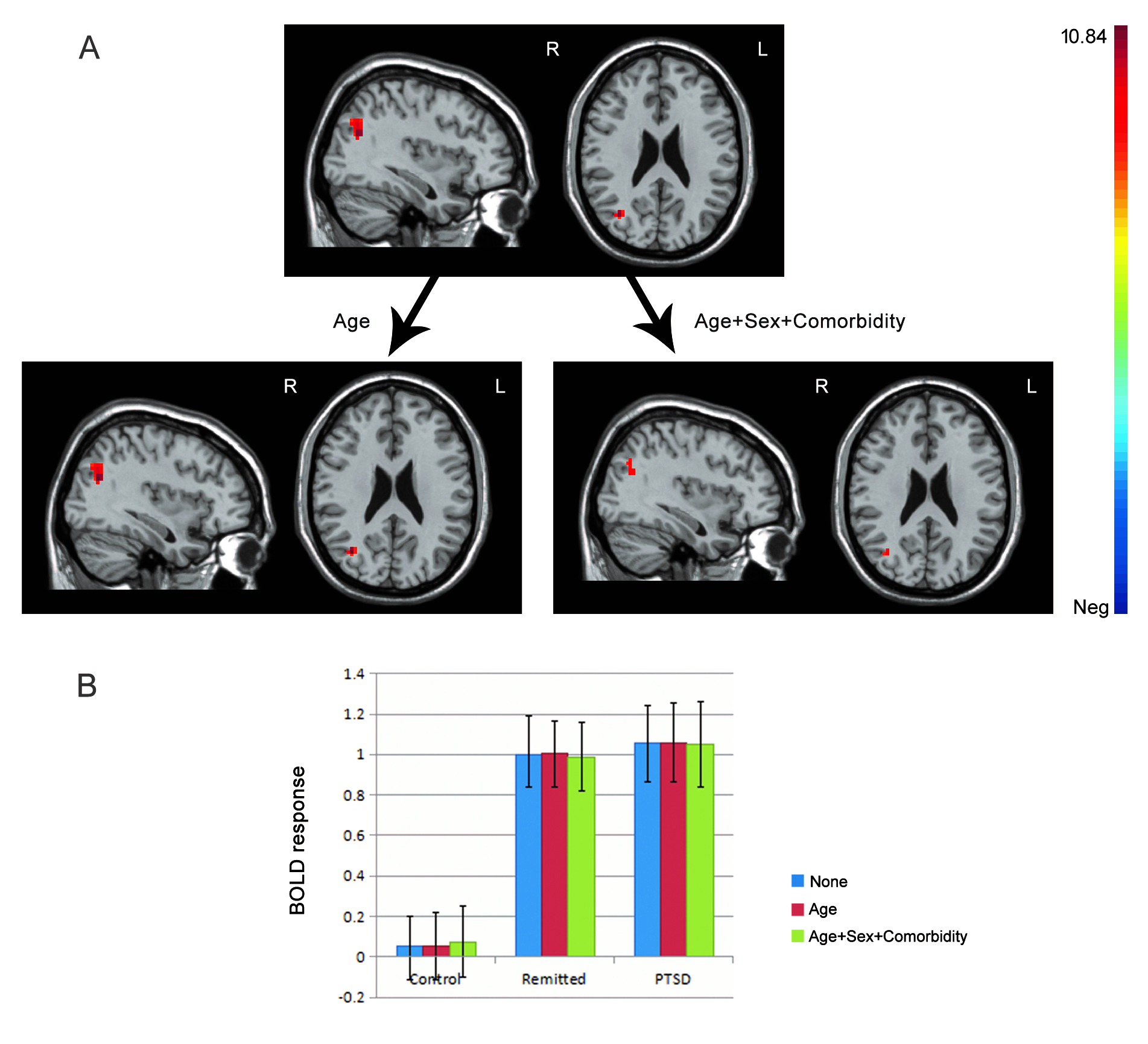

Supplement: Supplementary file 3 — Fig S2 [file BRB3-11-e02173-s004.tif]

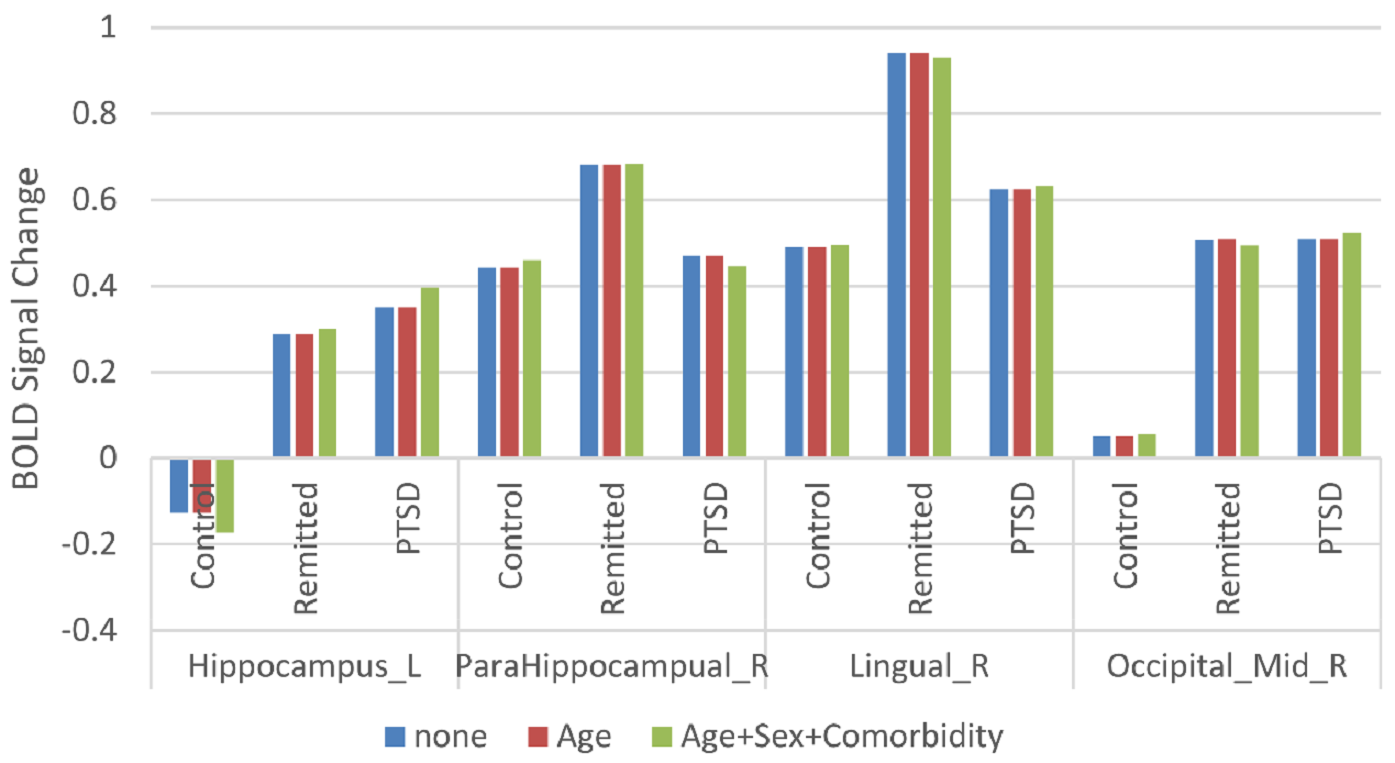

Supplement: Supplementary file 4 — Fig S3 [file BRB3-11-e02173-s005.tif]

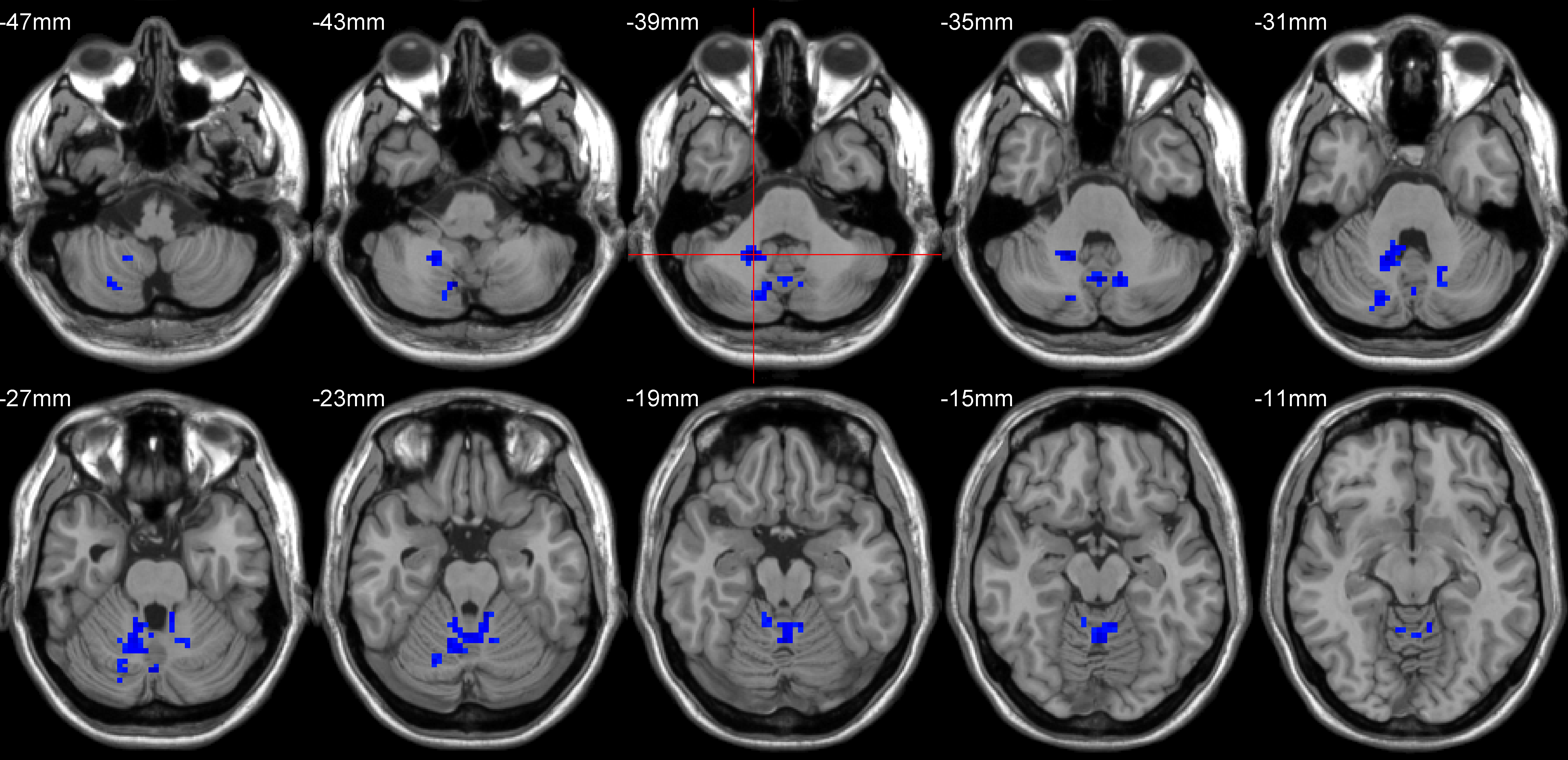

Supplement: Supplementary file 5 — Fig S4 [file BRB3-11-e02173-s001.tif]
